# Impact of Neurological Complications on Long-Term Outcomes in Patients with Infective Endocarditis

**DOI:** 10.3390/tropicalmed9060132

**Published:** 2024-06-13

**Authors:** Pedro Henrique Oliveira Murta Pinto, Isabela Galizzi Fae, Gustavo Brandão Oliveira, Roni Arley Silva Duque, Mauricio Vitor Machado Oliveira, Luan Salvador Machado Barbalho, André Oliveira Parreiras, Fernanda Alves Gelape, Fernanda Sophya Leite Cambraia, Guilherme Lelis Costa, Lucas Chaves Diamante, Renato Bráulio, Cláudio Léo Gelape, Andréa Teixeira-Carvalho, Teresa Cristina Abreu Ferrari, Maria Carmo Pereira Nunes

**Affiliations:** 1 Programa de Pós-Graduação em Ciências Aplicadas à Saúde do Adulto, Faculdade de Medicina da Universidade Federal de Minas Gerais, Avenida Professor Alfredo Balena, 190, Santa Efigênia, Belo Horizonte 30130-100, Minas Gerais, Brazil; phmurta@gmail.com (P.H.O.M.P.); isabelagalizzi@gmail.com (I.G.F.); brandaomed@hotmail.com (G.B.O.); tferrari@medicina.ufmg.br (T.C.A.F.); 2 Programa de Residência Médica em Cardiologia, Hospital das Clínicas da Universidade Federal de Minas Gerais, Avenida Professor Alfredo Balena, 110, Santa Efigênia, Belo Horizonte 30130-100, Minas Gerais, Brazil; roniduque@hotmail.com; 3 Departamento de Clínica Médica, Faculdade de Medicina da Universidade Federal de Minas Gerais, Avenida Professor Alfredo Balena, 190, Santa Efigênia, Belo Horizonte 30130-100, Minas Gerais, Brazil; maumauvitor@gmail.com (M.V.M.O.); luansmb8@gmail.com (L.S.M.B.); parreirasandre6@gmail.com (A.O.P.); fernandacambraia.br@gmail.com (F.S.L.C.); guilherme_lelis_@hotmail.com (G.L.C.); lucasdiamante02@gmail.com (L.C.D.); 4 Faculdade de Ciências Médicas de Minas Gerais, Alameda Ezequiel Dias, 275, Centro, Belo Horizonte 30130-110, Minas Gerais, Brazil; fernandagelape@hotmail.com; 5 Departamento de Cirurgia, Faculdade de Medicina da Universidade Federal de Minas Gerais, Avenida Professor Alfredo Balena, 190, Santa Efigênia, Belo Horizonte 30130-100, Minas Gerais, Brazil; renatobraulio1000@gmail.com (R.B.); clgelape@uol.com.br (C.L.G.); 6 Fundação Oswaldo Cruz, Instituto René-Rachou, FIOCRUZ Minas, Laboratório de Biomarcadores de Diagnóstico e Monitoração, Avenida Augusto de Lima, 1715, Barro Preto, Belo Horizonte 30190-002, Minas Gerais, Brazil; atcteixeira@gmail.com

**Keywords:** endocarditis, neurological, complications, long-term outcomes, prognosis

## Abstract

Neurological complications are frequent during the active course of infective endocarditis (IE), and they are associated with high in-hospital mortality rates. However, limited data exist on the prognostic value of these complications for late outcomes. This study aimed to assess the long-term impact of neurological complications in patients surviving an IE episode. A total of 263 consecutive IE patients admitted to a tertiary care center between 2007 and 2022 were prospectively included. Neurological complications at admission included transient ischemic attack (TIA), ischemic stroke, hemorrhagic stroke, intracerebral abscess, and meningitis. The primary outcome was a composite of overall mortality or heart valve surgery. Of the patients, 34.2% died in the hospital, leaving 173 survivors for long-term follow-up. Over a median of 3.5 years, 29 patients died, and 13 (9%) underwent cardiac surgery, resulting in an overall adverse event rate of 30%. Neurological complications independently predicted long-term adverse outcomes (hazard ratio (HR) 2.237; 95% CI 1.006–4.976), after adjusting for age, chronic kidney disease (CKD), and heart failure (HF) development. In an IE patient cohort, neurological complications at admission, which is a complication directly related to the IE process, were independent predictors of long-term outcomes.

## 1. Introduction

Infective endocarditis (IE) is a rare disease, with an estimated incidence of 6.3 to 15 cases per 100,000 people [1,2], but its incidence has been increasing in recent years [3,4]. Despite advances in diagnostic methods, medical treatment, and surgical procedures, IE still carries a poor prognosis. Neurological complications are frequent and impose a heavy burden on both in-hospital morbidity and mortality. In a previous study, 82% of the patients who underwent magnetic resonance imaging (MRI) were found to have ischemic brain lesions, intraparenchymal hemorrhage, subarachnoid hemorrhage, microhemorrhages, silent aneurysms, and brain abscesses [5]. Symptomatic neurological events may complicate up to 25% of IE cases, with the majority (86%) identified upon hospital admission or within the first week of antibiotic treatment [6]. 

Furthermore, patients remain susceptible to unfavorable outcomes after the initial IE episode [7,8,9]. Previous studies reported that repeat IE occurred in 4.8% of cases, with 81% considered reinfection and 19% relapses [10]. Ischemic and hemorrhagic stroke rates were 6.7% and 2.7%, respectively [7]. These rates appeared to be higher throughout the first year of follow-up [11] and among patients with repeated IE [7] or those who suffered a stroke during the index hospitalization [11]. Among patients for whom medical treatment was the initial strategy, surgery is needed in approximately 10% of cases, while for 6% of those who underwent surgical treatment during the first hospitalization, a late procedure may be recommended [8]. Mortality rates are high, reaching up to 24% at six months after discharge [9] and 45% in the long term [8].

While the predictors of in-hospital complications are relatively well defined, the factors that determine long-term outcomes in patients who had IE are not well established. Therefore, this study aims to assess the long-term impact of neurological complications at hospital admission in patients who survived an index IE episode. 

## 2. Materials and Methods

A total of 263 consecutive patients with IE admitted to a tertiary care center between 2007 and 2022 were prospectively included. Patients were classified as having either definite (68.3%) or possible IE (31.7%), according to the modified Duke criteria until 2015, and according to the European Society of Cardiology (ESC) modified criteria from 2015 on [12,13,14]. Native valve IE (NVIE), prosthetic valve IE (PVIE), and cardiovascular implantable electronic device (CIED)-IE patients, aged 18 years or older, were considered eligible for the study. Patients with isolated generator pocket infection, without involvement of the transvenous portion of the leads or of the tricuspid valve, were not included. The study was approved by the Ethics Committee of the Universidade Federal de Minas Gerais, Brazil (ETIC 412/06), and all patients signed the written informed consent form. 

Clinical characteristics (including predisposing cardiac conditions, systemic illnesses, main signs or symptoms attributed to IE, and related complications), laboratory tests (including C-reactive protein (CRP) and blood cultures), and echocardiographic results were recorded at hospital admission. All patients underwent either transthoracic or transesophageal echocardiography (TTE and TEE, respectively), as medically indicated, to define the presence, size, and location of vegetations and paravalvular complications. Cranial computed tomography (CT) or MRI, lumbar puncture, and abdominal imaging studies were not systematically performed. Patients with signs and symptoms suggestive of neurological complications or peripheral embolic events underwent such investigations. Moreover, patients who did not fulfil the diagnostic criteria for IE but were strongly suspected of having it underwent additional studies to explore the occurrence of vascular phenomena, which would be an additional minor criterion.

During treatment, complications such as embolic events, stroke, heart failure (HF), the need for cardiac surgery, or death were assessed. Lead extraction in CIED-IE was not considered cardiac surgery since it is the mainstay of the treatment. Data regarding valve tissue (from patients who underwent heart surgery) and extracted-lead cultures (from CIED-IE patients) were also recorded.

All patients who were discharged from the hospital were included in the present study. The data were collected preferably during follow-up visits at the institution’s outpatient clinic, carried out by the patient’s respective physician, every four months or more often (according to clinical status). The patients who did not show up had their data collected by means of phone contacts or through their medical records’ review.

Neurological complications at hospital admission were defined as transient ischemic attack (TIA), ischemic stroke, hemorrhagic stroke, intracerebral abscess, and meningitis. Nonspecific neurological symptoms were not considered. The primary outcome was a composite of overall mortality or heart valve surgery during follow-up. Patients who underwent heart valve surgery were censored at the time of the procedure.

Baseline characteristics were summarized as absolute numbers and frequencies for categorical variables and as the median value (and interquartile range) for the continuous ones. Cox regression models were used to determine the characteristics independently associated with the composite outcome. In the multivariate model, adjusting for age and sex, we included only pre-specified variables with known prognostic value. The variables selected for the model were age, sex, diabetes mellitus, chronic kidney disease (CKD), CRP, *Staphylococcus aureus* infection, neurological events, and HF. We compared the rates of death and cardiac surgery between patients with and without the independent predictors identified in the multivariate analysis using Kaplan–Meier survival curves. Statistical significance was assumed at *p* < 0.05. The analysis was performed using the software IBM SPSS Statistics for Windows, version 26.0 (Armonk, NY, USA: IBM Corp).

The funding source had no involvement in the study design, data collection, analysis, and interpretation, or in the writing of the paper.

## 3. Results

### 3.1. Baseline Characteristics

Patients’ baseline characteristics at hospital admission are presented in Table 1. The majority of patients had NVIE (47.5%), 23.1% had PVIE, 21.7% had CIED-IE, and 66.5% were classified as having left-sided IE. Rheumatic heart disease (RHD) was the most frequent cardiac predisposing condition (30.2%). Other identified conditions included mitral valve prolapse (MVP) in 11.4%, degenerative valve disease in 11.6%, and congenital heart disease in 9%, while 8.5% of the patients had a history of a previous episode of IE. The main comorbidities were diabetes mellitus (14.2%) and CKD (12.9%). Human immunodeficiency virus (HIV) infection was observed in only 0.9% of the cases and intravenous (IV) drug abuse in 1.2%.

### 3.2. Complications at Presentation

At hospital admission, 18.2% of the patients were diagnosed with a symptomatic neurological complication; most (60%) of them suffered ischemic events (TIA or stroke), 18% experienced a hemorrhagic stroke, and 22% had either intracerebral abscesses or meningitis. Neurological complications were significantly more frequent among those with left-sided IE (23.4%) compared to those with right-sided IE (5.7%) (*p* = 0.008). As far as other types of complications are concerned, 13.1% of the patients experienced systemic embolic events and 27% had paravalvular lesions. Table 2 highlights the differences in the baseline characteristics between patients with and without neurological complications. 

### 3.3. Other Characteristics during Treatment

*Staphylococcus aureus* was the most common causative microorganism, followed by coagulase-negative staphylococci, *Streptococcus* sp., *Enterococcus* sp., and non-HACEK (*Haemophilus* spp., *Actinobacillus* spp., *Cardiobacterium hominis*, *Eikenella* spp., and *Kingella* spp.) Gram-negative bacteria. IE due to HACEK was diagnosed in only one patient (0.4%), and nine (3.4%) had fungal IE. In the subgroup of patients who presented with neurological complications, the most common causative microorganism was also *S. aureus* (29.1%).

After the initiation of antibiotic treatment, the most common complication was heart failure (HF). Heart valve surgery was performed in 57.5% of the patients, surgical mortality was 31.8%, and overall in-hospital mortality was 34.2%. Of those who died, 22.9% had suffered a neurological complication at admission. Table 3 highlights the differences in the baseline characteristics between patients who survived and those who died during hospital stay. 

### 3.4. Long-Term Outcomes

**Figure 1:**
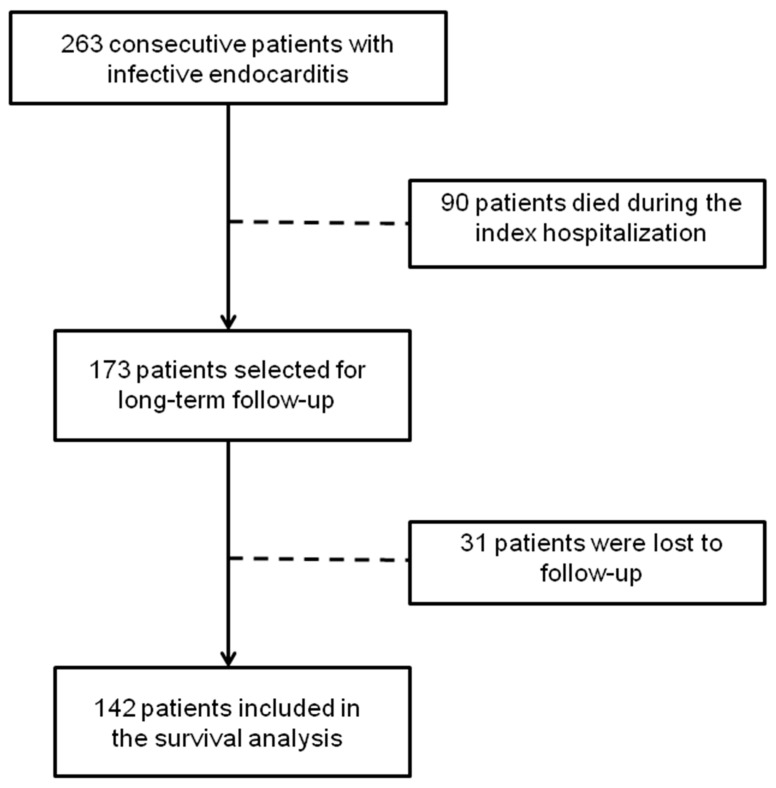
Study flow chart.

Among the 173 survivors discharged from the hospital, 31 were lost to follow-up (17.9%). Thus, information about major adverse events was available for the remaining 142 patients (Figure 1). During a mean follow-up of 4.8 years (median of 3.5 years, range 2 months to 16.8 years), the primary composite endpoint was reached in 42 patients (29.5%). The overall incidence of adverse events was 6.14 per 100 patient-years. The overall mortality rate was 20%; subsequent cardiac surgery for valve replacement was performed in 13 (9%) patients, 38% of whom had already undergone surgery during the index hospitalization. During the follow-up period, a new IE episode was observed in five patients and symptomatic ischemic stroke in another five.

**Figure 2:**
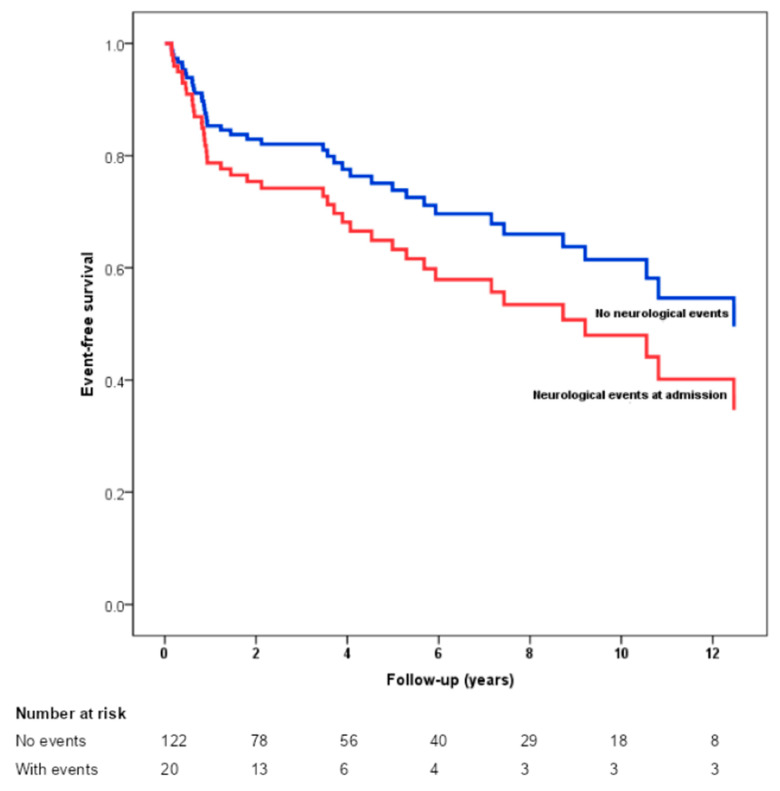
Kaplan–Meier estimates of event-free survival according to neurological complications at hospital admission.

Table 4 shows the factors associated with the primary outcome in univariable and multivariable analysis. Age, neurological complications at hospital admission, chronic kidney disease (CKD) at baseline, and the development of heart failure (HF) during treatment were identified as independent predictors of death and the need for heart valve surgery in the long-term follow-up. Other variables that express disease severity at admission (including CRP levels, *Staphylococcus aureus* as the causative microorganism, and large dimensions of vegetation) were associated with in-hospital mortality but not with long-term outcomes. Kaplan–Meier curves show significant differences in event-free survival according to neurological complications at admission (Figure 2).

## 4. Discussion

IE is a severe condition with high morbidity and mortality, and its predictors of in-hospital adverse outcomes have been well described in previous studies [15,16,17,18]. Moreover, the risk of death and other cardiovascular events remains higher than in the general population even after the completion of treatment and hospital discharge [7]. Neurological complications are the most frequent extracardiac manifestations of IE, affecting around 25% to 70% of individuals with the condition [19]. 

In this study, we evaluated the incidence of neurological complications and other adverse events and their predictors in patients who survived an index IE episode for a median follow-up period of 3.5 years. The primary endpoint of overall mortality or the need for cardiac surgery occurred in 42 patients (30%), with an overall incidence of adverse events of 6.14 per 100 patient-years. Patients who presented with neurological complications at hospital admission had twice as high a risk of developing a primary outcome event as those who did not. Higher age, CKD at baseline, and HF development during treatment were also identified as independent predictors of late adverse events.

García-Cabrera et al. previously analyzed data from a multicenter cohort of patients with IE and found that 25% of them experienced neurological complications (ischemic stroke being the most frequent at 56%), conferring a higher risk of death during the hospital stay or up to one month after discharge (45% versus 24%). Patients at the highest risk of developing neurological complications were those with mitral valve IE, *S. aureus* infection, larger vegetation length, and those on anticoagulant therapy before the onset of the IE episode. However, even though the patients were followed up for a year after discharge and the deferred death rates were analyzed, the association of neurological complications with this outcome was not investigated. Moreover, one must acknowledge that neurological complications were present at hospital admission in only 7% of the total cohort, and patients who developed this complication even later than four weeks after the start of the antibiotic treatment were also included [6].

More recently, Ching-Chang et al. addressed this same question in a 12-year, nationwide cohort of IE patients in Taiwan, retrospectively analyzing data from the National Health Insurance Research Database. Among the entire cohort, 6.27% of the patients had neurological complications, present at hospital admission or occurring within up to three months after discharge, and they had significantly higher mortality rates compared to those who did not show this type of complication (nearly 50% over the five-year follow-up period) [20].

Our study is a cohort of consecutive patients admitted to a tertiary-care, university-based hospital responsible for the care of patients in a large area of the state. Patients showed a high rate of symptomatic neurological complications at hospital admission (18.2%), expressing the disease severity expected from patients referred to this type of center. As opposed to previous studies [6,20], in which either patients were not followed for longer than one year or they were retrospectively included, we prospectively investigated the relationship of neurological complications with mortality and the need for late valve surgery in the long-term follow-up (median 3.5 years). Our results reinforce the great impact that neurological complications have on IE patients’ prognosis, and since we specifically considered the neurological complications diagnosed at hospital admission as a variable of interest, this should be interpreted as a strong indicator of poor prognosis and the need for more aggressive treatment early in the course of the disease.

The underlying pathophysiological mechanisms linking IE to neurological complications include bacterial embolization, septic emboli, immune-mediated mechanisms, and cerebral microvascular damage. These complications often manifest as embolic stroke, frequently presenting as initial symptoms of IE. However, the clinical spectrum of neurological involvement is diverse, encompassing ischemic or hemorrhagic stroke, infected intracranial aneurysm, meningitis, brain abscess, spinal epidural abscess, encephalopathy, mononeuropathy, and seizures. Conversely, neurological complications may remain asymptomatic, with imaging studies revealing evidence of underlying disease in 30% [21] to 82% of IE cases [5]. The risk of developing neurological complications in IE is primarily influenced by the characteristics of the vegetations and the duration of antibiotic therapy. Larger vegetations, particularly those located on the left side of the heart, are more prone to embolization, with this risk heightened before the initiation of antibiotic treatment or within the initial week of therapy. Furthermore, anticoagulant use at presentation is associated with an increased risk of hemorrhagic complications. Therefore, neurological assessments to detect complications are essential for patient management and risk stratification. 

According to the study by Shih et al., ischemic stroke was detected in 6.7% of the patients during the follow-up period [7]. Additionally, patients who had experienced neurological complications during the index hospitalization had a higher risk of a second neurological event during the first year after hospital discharge [11]. In the present study, 3.5% of the survivors suffered a symptomatic ischemic stroke and none of them suffered a hemorrhagic stroke during the follow-up.

In the present study, CKD was found to be a strong independent predictor of long-term mortality and the need for surgical treatment. Whether this is solely due to the increased risk of death attributed to CKD itself or if there is an interaction between CKD and the IE episode is not possible to ascertain. It is well known that patients with CKD have an increased risk of overall and cardiovascular mortality over time [22]. However, a large cohort by Pericàs et al. [23], which compared in-hospital and six-month outcomes in IE patients with and without CKD on hemodialysis (HD), showed that patients on HD have higher in-hospital and six-month mortality rates, in addition to a higher risk of relapses. The risk factors associated with the increased six-month mortality in the previous cohort of HD patients were Charlson score [24], central nervous system emboli and other systemic emboli, persistent bacteremia, and acute heart failure. Furthermore, patients on HD had significantly more NVIE, mitral valve involvement, and vegetation identified on echocardiogram when compared to those who were not on HD, suggesting that there are specific mechanisms of disease severity in the former subgroup of patients. Even though patients with CKD not on HD were not included, a comparison between HD patients and end-stage renal disease patients not on HD did not show any significant differences regarding stroke, systemic embolization, in-hospital mortality, six-month mortality, surgery, and relapses. The aforementioned data corroborate our findings [23].

Acute or worsening HF during treatment is one of the most severe complications of IE, and the strongest recommendation for emergent surgery during hospital stay, according to current guidelines [25]. Park et al. developed a model to predict six-month mortality in IE, in which NYHA class III or IV HF figured as an independent predictor [9]. Surgical mortality in IE patients is higher than in heart valve surgery patients without IE. Despite this, surgical treatment has shown a beneficial effect on long-term mortality in previously published studies [8,9,26,27]. In our study, the primary endpoint incidence was significantly higher in the patients who developed HF during treatment. Surgical treatment during the index hospitalization, however, was not associated with a lower risk of death after hospital discharge.

The rate of relapse or reinfection reported in the literature is 4.8% to 11.7% [7,10]; the majority of cases are reinfections (81%) [28] and occur within the first year of follow-up [7]. Furthermore, one-year survival is lower in patients who experience a second IE episode [28]. In our study, the rate of repeated IE was 3.5%. One reason that could explain why this rate was lower than previously described is the fact that the prevalence of IV drug abuse, a known risk factor for EI repetition, in our population was only 1.2%.

Our study has several strengths. It is a cohort of patients with IE in a low-to-middle-income country (LMIC), and this is very representative since our population shows different characteristics than those of the studies previously published. The median age of our patients is lower, and this is probably due to the higher prevalence of RHD, which is the most common predisposing condition (as opposed to degenerative valve disease in high-income countries). Another difference is the substantially higher proportion of patients with CIED-related IE in our population. This is in agreement with a recent study that showed a significant increase in CIED-IE from 1998 to 2013 [29]. This may also be explained by the fact that our institution is the referral center for device implantation in the public health system in a large area of our state, with a high volume of procedures per year. The main causative microorganism identified, however, was not *Streptococcus* sp., as it would be expected in an LMIC according to previous studies [30]. This may represent a shift in epidemiology to a pattern closer to the one in high-income countries.

This study has some limitations. Patients with possible IE were included, and this was due to the high percentage of culture-negative IE. The fact that our patients were not systematically submitted to brain imaging studies, but only when medically recommended for the investigation of symptoms, did not allow for evaluating neurological involvement without symptoms. Hence, the rate of overall neurological events is probably underestimated. Nonetheless, it is controversial whether asymptomatic findings on neuroimaging are predictors of poorer outcomes [5], whereas our results reinforce that symptomatic events alone actually are. The population was heterogeneous, including NVIE, PVIE, and CIED-IE. There was a 17.9% loss to follow-up, which might underestimate the primary outcome rate during follow-up. This may be a reason why our mortality rate was lower than that in previous studies. We also acknowledge that the number of patients with neurological complications in the follow-up cohort might affect the reliability of the Kaplan–Meier curve (Figure 2) beyond two years. This should be considered when interpreting the long-term results, and further studies with larger sample sizes are needed for more robust conclusions. Additionally, some patients had their data collected from medical record review, and we did not compare patients with previous IE with controls.

This study reinforces the importance of recognizing symptomatic neurological complications at hospital admission as a strong predictor of poor prognosis. Additionally, it highlights the importance of identifying this specific patient subgroup as one that may derive potential benefits from extended follow-up visits beyond one year post hospital discharge, as advised by current guidelines [25]. Future studies should address whether an approach with more aggressive treatment for patients with the aforementioned complications could further reduce the rate of long-term complications.

## 5. Conclusions

In a large cohort of patients with prior IE, it was observed that neurological complications occurring at hospital admission, which are complications directly related to the IE process, were identified as strong independent predictors of overall mortality and the need for cardiac surgery. These findings highlight the significant impact of neurological complications on the patients’ overall long-term prognosis. By recognizing the association between neurological complications and adverse outcomes, this study may contribute to the understanding of IE management and potentially improve patient care.

## Figures and Tables

**Table 1:** IE patient characteristics at hospital admission.

Characteristics	
Age (years)	52 [36–64]
Male sex	159 (60.5)
Predisposing condition	
Previous IE	22 (8.5)
Rheumatic heart disease	79 (30.2)
Prosthetic valve	68 (26.1)
CIED	77 (29.3)
Diabetes mellitus	37 (14.2)
Chronic kidney disease	34 (12.9)
Laboratory findings	
Hemoglobin (g/dL)	10.1 [8.7–11.7]
Leukocytes (cells ×10^3^/µL)	11.1 [7.7–15.8]
NLR	5.6 [3.2–10.3]
CRP (mg/L)	73 [32.6–177]
Main causative microorganisms	
*Staphylococcus aureus*	54 (20.5)
Coagulase-negative staphylococci	38 (14.4)
Streptococci	37 (14.1)
*Enterococcus* sp.	20 (7.6)
Gram-negative bacteria	13 (4.9)
Negative culture	89 (33.8)
Echocardiographic findings	
Vegetation size	11 [8–15]
Paravalvular leak, leaflet perforations, chordae tendinea rupture, mitral-aortic intervalvular fibrosa abscess	71 (27)

Data are expressed as median [interquartile range], or absolute numbers (percentage). IE: infective endocarditis; CIED: cardiovascular implantable electronic device; NLR: neutrophil-to-lymphocyte ratio; CRP: C reactive protein.

**Table 2:** Characteristics at hospital admission of patients with and without neurological complications.

Characteristics	Patients with NC at Hospital Admission (n = 48)	Patients without NC at Hospital Admission (n = 215)	*p* Value
Age (years)	52 [37–63]	52 [36.2–64.0]	0.629
Male sex	28 (58.3)	131 (60.9)	0.755
Predisposing condition			
Previous IE	3 (6.2)	19 (8.8)	0.732
Rheumatic heart disease	14 (29.1)	65 (30.2)	0.865
Prosthetic valve	10 (20.8)	58 (26.9)	0.325
CIED	8 (16.6)	69 (32.0)	0.025
Diabetes mellitus	8 (16.6)	29 (13.4)	0.772
Chronic kidney disease	3 (6.2)	31 (14.4)	0.234
Laboratory findings			
Hemoglobin (g/dL)	9.7 [8.9–10.9]	10.1 [8.6–11.7]	0.274
Leukocytes (cells ×10^3^/µL)	12.7 [8.7–18.9]	10.7 [7.1–14.5]	0.039
CRP (mg/L)	82 [51.2–181]	68.4 [30–172.8]	0.136
Main causative microorganisms			
*Staphylococcus aureus*	14 (29.1)	40 (18.6)	0.107
Coagulase-negative staphylococci	3 (6.2)	35 (16.2)	0.075
Streptococci	10 (20.8)	27 (12.5)	0.210
*Enterococcus* sp.	5 (10.4)	15 (6.9)	0.407
Gram-negative bacteria	1 (2.0)	12 (5.5)	0.315
Negative culture	13 (27.0)	76 (35.3)	0.285
Echocardiographic findings			
Vegetation size	12 [7–16]	11 [8.0–15.0]	0.895
Paravalvular leak, leaflet perfora- tions, chordae tendinea rupture, mitral-aortic intervalvular fibrosa abscess	14 (29.1)	57 (26.5)	0.793

Data are expressed as median [interquartile range], or absolute numbers (percentage). NC: neurological complications; IE: infective endocarditis; CIED: cardiovascular implantable electronic device; CRP: C reactive protein.

**Table 3:** Characteristics at hospital admission of patients who survived versus those who died during hospital stay.

Characteristics	Survivors (n = 173)	Deceased (n = 90)	*p* Value
Age (years)	51 [33.5–63]	54 [40.7–66.0]	0.073
Male sex	107 (61.8)	52 (57.8)	0.522
Predisposing condition			
Previous IE	14 (8.1)	8 (8.9)	0.805
Rheumatic heart disease	53 (30.6)	26 (28.9)	0.863
Prosthetic valve	48 (27.7)	20 (22.2)	0.335
CIED	54 (31.2)	23 (25.5)	0.416
Diabetes mellitus	19 (11.0)	18 (20.0)	0.040
Chronic kidney disease	22 (12.7)	12 (13.3)	0.919
Laboratory findings			
Hemoglobin (g/dL)	10.3 [8.7–12.0]	9.4 [8.3–11.2]	0.689
Leukocytes (cells ×10^3^/µL)	10.2 [7.0–14.4]	13.3 [9.6–17.9]	0.001
CRP (mg/L)	56 [25.4–135]	118 [55–242.0]	<0.001
Main causative microorganisms			
*Staphylococcus aureus*	28 (16.2)	26 (28.9)	0.014
Coagulase-negative staphylococci	25 (14.5)	13 (14.4)	0.973
Streptococci	30 (17.3)	7 (7.8)	0.037
*Enterococcus* sp.	13 (7.5)	7 (7.8)	0.919
Gram-negative bacteria	8 (4.6)	5 (5.6)	0.726
Negative culture	64 (37.0)	25 (27.8)	0.150
Echocardiographic findings			
Vegetation size	10 [7–15]	14 [9.0–20.0]	0.008
Paravalvular leak, leaflet perforations, chordae tendinea rupture, mitral-aortic intervalvular fibrosa abscess	46 (26.6)	25 (27.8)	0.837

Data are expressed as median [interquartile range], or absolute numbers (percentage). IE: infective endocarditis; CIED: cardiovascular implantable electronic device; CRP: C reactive protein.

**Table 4:** Cox regression model for the primary endpoint of death or cardiac surgery in patients with previous IE.

Characteristics	Univariate Analysis		Multivariate Analysis	
	HR (95% CI)	*p* Value	HR (95% CI)	*p* Value
Age (years)	1.024 (1.005–1.043)	0.014	1.026 (1.006–1.047)	0.012
Male gender	1.65 (0.90–3.00)	0.102		
Rheumatic heart disease	1.03 (0.50–2.00)	0.924		
Degenerative valve disease	1.51 (0.60–3.43)	0.316		
Mitral valve prolapse	1.34 (0.56–3.20)	0.510		
Prosthetic valve	1.03 (0.51–2.07)	0.925		
Congenital heart disease	0.04 (0.001–3.31)	0.154		
Previous IE	1.01 (0.31–3.28)	0.982		
Diabetes mellitus	2.04 (0.77–5.35)	0.146		
Chronic kidney disease	2.95 (1.46–5.92)	0.002	4.265 (2.035–8.938)	<0.001
C-reactive protein	1.00 (0.99–1.00)	0.327		
*S. aureus* infection	1.30 (0.60–2.82)	0.505		
Vegetation size	0.99 (0.92–1.05)	0.749		
Paravalvular complications	1.04 (0.52–2.07)	0.910		
Neurological events ^a^	1.50 (0.69–3.26)	0.297	2.237 (1.006–4.976)	0.048
Embolic events	1.60 (0.56–4.52)	0.370		
Heart failure ^b^	1.95 (1.04–3.64)	0.036	1.982 (1.035–3.795)	0.039
Cardiac surgery	0.84 (0.44–1.59)	0.603		

^a^—at hospital admission. Transient ischemic attack (TIA), ischemic stroke, hemorrhagic stroke, intracerebral abscess, or meningitis. ^b^—developed during IE treatment. HR: hazard ratio; CI: confidence interval; CIED: cardiovascular implantable electronic device; CRP: C-reactive protein; IE: infective endocarditis.

## Data Availability

The datasets presented in this article are not readily available due to privacy and ethical reasons. Requests to access the datasets should be directed to M.C.P.N.; mcarmo@waymail.com.br.
